# Optimization of Layered Dissolving Microneedle for Sustained Drug Delivery Using Heat-Melted Poly(Lactic-Co-glycolic Acid)

**DOI:** 10.3390/pharmaceutics13071058

**Published:** 2021-07-10

**Authors:** Chisong Lee, Jinkyung Kim, Daniel Junmin Um, Youseong Kim, Hye Su Min, Jiwoo Shin, Jee Hye Nam, Geonwoo Kang, Mingyu Jang, Huisuk Yang, Hyungil Jung

**Affiliations:** 1 Department of Biotechnology, Yonsei University, Seoul 03722, Korea; lchs0625@yonsei.ac.kr (C.L.); jinkung1324@naver.com (J.K.); danny0619@yonsei.ac.kr (D.J.U.); ustarkim@yonsei.ac.kr (Y.K.); vitamin94@yonsei.ac.kr (H.S.M.); jiwooshin@yonsei.ac.kr (J.S.); jeehyenam@yonsei.ac.kr (J.H.N.); gwkang@juvicbio.com (G.K.); 2 Juvic Inc., 272 Digital-ro, Guro-gu, Seoul 08389, Korea; mgjang@juvicbio.com (M.J.); hsyang@juvicbio.com (H.Y.)

**Keywords:** poly(lactic-co-glycolic acid), dissolving microneedle, sustained release, melting method, thermoplastic characteristics, drug delivery system

## Abstract

Dissolving microneedles (DMNs) have been used as an alternative drug delivery system to deliver therapeutics across the skin barrier in a painless manner. In this study, we propose a novel heat-melting method for the fabrication of hydrophobic poly(lactic-co-glycolic acid) (PLGA) DMNs, without the use of potentially harmful organic solvents. The drug-loaded PLGA mixture, which consisted of a middle layer of the DMN, was optimized and successfully implanted into ex vivo porcine skin. Implanted HMP-DMNs separated from the patch within 10 min, enhancing user compliance, and the encapsulated molecules were released for nearly 4 weeks thereafter. In conclusion, the geometry of HMP-DMNs was successfully optimized for safe and effective transdermal sustained drug delivery without the use of organic solvents. This study provides a strategy for the innovative utilization of PLGA as a material for transdermal drug delivery systems.

## 1. Introduction

Dissolving microneedles (DMNs) have been developed to pierce the skin barrier and release encapsulated therapeutics into the epidermal layer in a minimally invasive manner [1,2,3]. After penetrating the skin, the biodegradable backbone polymers are dissolved inside the skin tissue by interstitial fluid, resulting in the release of the encapsulated molecules without any external stimulus [4,5]. DMNs provide potentially painless drug administration and lessen the side effects of hypodermic injection, such as hazardous waste and the probability of infection caused by reused needles [6,7,8]. Based on these advantages, DMNs have been widely researched in the fields of diabetes, vaccinations, and cancer therapy [9,10,11]. Hydrophilic polymers such as sodium hyaluronate (HA), carboxymethyl cellulose, and polyvinyl pyrrolidone have been widely used for generating the DMN matrix, because they allow for an easy fabrication process [12]. Although these hydrophilic polymer-based DMNs are suitable for the burst release of drugs on the basis of their rapid dissolution in the interstitial fluid, they are limited in releasing long-acting drugs, ones that are intended to be released for a few days or weeks, in a sustained manner. Therefore, in the case of targeting diseases that require continuous medication, such as chronic pain, hypertension, or alopecia areata, hydrophilic polymers are not suitable for the generation of the DMN matrices.

Because the dissolution characteristics of polymer-containing DMNs are critical to controlling the drug release profile, the selection of a matrix polymer suitable for a required duration for medication is critical in the fabrication of DMNs [2]. Therefore, hydrophobic polymers such as poly(lactic-co-glycolic acid) (PLGA) or polycaprolactone (PCL) have been widely used for DMN fabrication, to deliver long-acting therapeutics in a sustained manner [13,14]. A representative approach tested PLGA as a matrix polymer for DMNs, wherein various therapeutics such as methotrexate, levonorgestrel, or etonogestrel were encapsulated in PLGA DMNs [15,16,17]. These studies have successfully achieved sustained transdermal drug release from the PLGA matrix itself, for a few days to weeks after DMN implantation into the skin. However, for the fabrication of the DMNs, the preparation of the liquefied viscous mixture of the drug and matrix polymer requires it to be shaped according to the DMNs [18,19,20]. Because PLGA exists in a solid form, organic solvents have been used in most previous studies to fabricate PLGA-based DMNs [14,21].

Because organic solvents might be harmful when they are incompletely evaporated, an additional process to remove any residual organic solvent is necessary in the case of hydrophobic polymer-based DMNs [22]. To overcome this limitation, a recent study used heat-melted PCL rather than the dissolved form in organic solvents to fabricate DMNs for sustained capsaicin release [13]. Although not using the residual organic solvent certifies safety, controlling the desired drug release profile while using PCL that has sufficient mechanical strength for skin piercing when used in a DMN might be difficult, because the mechanical strength of PCL varies depending on its molecular weight [23]. Therefore, to achieve an ideal DMN system for sustained drug release using a hydrophobic polymer and a solvent-free DMN fabrication process, there is a need for sufficient mechanical strength of the fabricated DMNs regardless of the properties of the polymer. In addition, the versatility of the encapsulated drug in terms of hydrophilicity should be offered by excluding the use of an organic solvent.

In this study, a novel multilayered DMN patch was developed using heat-melted PLGA (HMP-DMN patch). The HMP-DMN was composed of three layers: the tip, middle, and base layers. The tip and base layers were fabricated with HA to provide sufficient mechanical strength and rapid separation characteristics, respectively, owing to the hydrophilic characteristics of HA. The middle layer consisted of heat-melted PLGA, which encapsulated the hydrophilic model dye rhodamine B (Rho B). Because PLGA was liquefied using heat rather than an organic solvent, based on the thermoplastic characteristics of PLGA, hydrophilic molecules with certified heat tolerance can be easily homogenized into the PLGA matrix regardless of their hydrophilicity. The fabrication process of the HMP-DMN array was optimized, with various proportions of each layer, to achieve perfect geometry for skin penetration and effective drug delivery. After optimization, the physical properties, skin penetration, and release profile of the HMP-DMNs were evaluated. When the optimized HMP-DMN penetrated the skin barrier, the base layer quickly dissolved with the interstitial fluid, and the PLGA middle layer implanted into the skin tissue after separation from the base layer. HMP-DMN showed a sustained release profile for 4 weeks, demonstrating its potential for application in the delivery of long-acting therapeutics including levonorgestrel, ibuprofen, or progesterone, if appropriate further studies are conducted with relevant model drug and in vivo evaluations. [15,24,25]. Furthermore, the simple separation of the HMP-DMNs would offer patient compliance, in the case of long-term medications, without the need to wear a patch for the entire period. In this study, the PLGA-based HMP-DMN patch was fabricated without using an organic solvent, and the DMN geometry was successfully optimized for safe and effective transdermal sustained drug delivery. This novel multilayered HMP-DMN patch fabricated without an organic solvent will allow for the innovative utilization of PLGA as a safe and effective transdermal drug delivery system.

## 2. Materials and Methods

### 2.1. Preparation of Heat-Melted PLGA

PLGA was selected as a matrix polymer for the HMP-DMN patch because of its biocompatibility, slow degradation, and the advantage of controlling its degradation time by tuning the ratio of copolymers, lactic acid, and glycolic acid [26]. PLGA (50:50 molar ratio of lactic acid and glycolic acid; inherent viscosity: 0.59 dL/g) (Sigma-Aldrich, St. Louis, MO, USA) was melted on a hot plate stirrer (MSH-20D, Daihan Scientific Co., Seoul, Republic of Korea) at 130 °C. Following this, Rho B (Sigma-Aldrich) was mixed with PLGA (99:1 weight ratio of PLGA/Rho B). The liquid-state mixture was placed in a syringe covered with an air-type Peltier temperature control unit (TB-10E-K, Musashi Engineering Inc., Tokyo, Japan). The temperature of the syringe was kept at 100 °C to maintain the liquid state.

### 2.2. Fabrication of the HMP-DMNs

Arrays of 3 × 3 microneedle master structures were prepared from stainless-steel using the laser cutting method, with a height and base diameter of 800 μm and 450 μm, respectively. A polydimethylsiloxane (PDMS) prepolymer base, Sylgard 184A, and curing agent, Sylgard 184 B (Dow Corning, Midland, MI, USA), were thoroughly mixed in a weight ratio of 10:1 and poured into the master structures. The prepolymer mixture was placed in a vacuum for 0.5 min to remove all the air bubbles trapped in the inverse-PDMS mold and cured at 80 °C for 3 h. The resulting PDMS mold layers were peeled off the master structures. These molds were utilized for the fabrication of the HMP-DMNs. The conical cavities in the molds were arranged in 3 × 3 arrays with 1.5 mm space in an area of 3.45 mm^2^, with a 450 µm base diameter and 800 µm height.

Two casting solutions were used as the backbone to make the HMP-DMNs. The first solution was prepared by mixing PLGA and Rho B for sustained release. The second HA (30 kDa, PrimalHyal50; Soliance, Pomacle, France) solution of 60% (*w/v*) was prepared using a planetary centrifugal mixer (ARV-310, Thinky Corp., Tokyo, Japan) at 5000 rpm for 30 min. HA was selected based on a biodegradable, biocompatible, and viscous nature [27]. The PLGA and Rho B solution, which was heated using a temperature control unit to 100 °C to maintain the liquid state, was dispensed as an array of 3 × 3 droplets into the cavity of the mold using a robotic dispenser (SHOT mini 100S, Musashi Engineering Inc.). This dispensing process was performed with three different dispensing amounts of PLGA mixtures, which were controlled by altering the dispensing times, 0.1, 0.2, and 0.3 s, at a pressure of 250 kPa. After dispensing the PLGA mixtures, HA solution was added into the mold and centrifuged for three different centrifugation times (10, 20, and 30 min) at 3470× *g* [28]. Images of the fabricated HMP-DMNs were acquired using a stereomicroscope (M165FC, Leica Camera AG, Wetzlar, Germany) and a digital microscope camera (DFC450C, Leica Camera AG).

### 2.3. Skin Penetration Analysis of the HMP-DMNs

To evaluate skin penetration, an HMP-DMN patch with fluorescent Rho B dye was inserted into stretched pig cadaver skin (Cronex, Hwaseong-si, Korea) using thumb force for 10 s. After the DMN patch was detached from the pig cadaver skin, the surface of the skin was dyed with 0.4% Trypan Blue solution (Sigma-Aldrich) for 30 min. After washing the superfluity of the Trypan Blue solution on the porcine skin with distilled water, stained pig cadaver skins were observed using a stereomicroscope and a digital microscope camera to visualize the HMP-DMN penetration.

### 2.4. Separation Analysis of the HMP-DMNs

After application of the HMP-DMN patch into stretched pig cadaver skin with thumb force for 10 s, the patch was detached from the skin every 5 min for 10 min. The skin containing the separated PLGA mixture and the residual on the patch after application were observed using a stereomicroscope and a digital microscope camera. The separation efficacy of HMP-DMNs from the patch was evaluated by observing the dye-loaded PLGA layers in the HMP-DMN patch before and after skin insertion.

### 2.5. Fracture Force Analysis of the HMP-DMNs

The mechanical properties of the HMP-DMNs were evaluated using a force analyzer (Z0.5TN, Zwick Roell Inc., Ulm, Germany). A single HMP-DMN was placed on the test stage positioned vertically, while a metal probe moved vertically downward at a continuous speed of 3.6 mm/min. The initial distance between the probe and stage was 1.5 mm. After the probe reached the tip of the HMP-DMN, the force was gradually increased 0.02 N/s up to a maximum force of 5 N. When a fracture occurred in the HMP-DMN, the fracture force was recorded.

### 2.6. In Vitro Release Profile Analysis of the HMP-DMNs

To estimate the release profile of Rho B from the HMP-DMN patch, phosphate-buffered saline (PBS, pH 7.4; Life Technologies, Carlsbad, CA, USA) containing 25% (*v/v*) ethanol was used as the releasing medium [29]. The HMP-DMN patch was fabricated, and the amount of Rho B encapsulated in the 3 × 3 arrays of the HMP-DMN was calculated using the following formula:

Amount of Rho B (mg) = [weight of mold with 9 droplets (mg) − weight of mold (mg)] × 1/100

The control DMN patches were fabricated using 60% (*w/v*) HA solution containing Rho B by carrying out a round of centrifugation at 3470× *g* for 2 min without PLGA. The amount of Rho B in the control DMN patch was the same as that in the HMP-DMN patch. To analyze the release of Rho B, control DMN patches and HMP-DMN patches were placed in 5 mL of PBS in a conical tube and then placed on a hot plate stirrer at 37 °C and 100 rpm for 26 days. Every 2 days, the released PBS was replaced with 5 mL of fresh and preheated medium. The replaced PBS was analyzed using a multimode plate reader (VICTOR™ X, PerkinElmer, Waltham, MA, USA) to estimate the amount of Rho B in each patch.

## 3. Results

### 3.1. Fabrication of the HMP-DMN Patch

**Figure 1:**
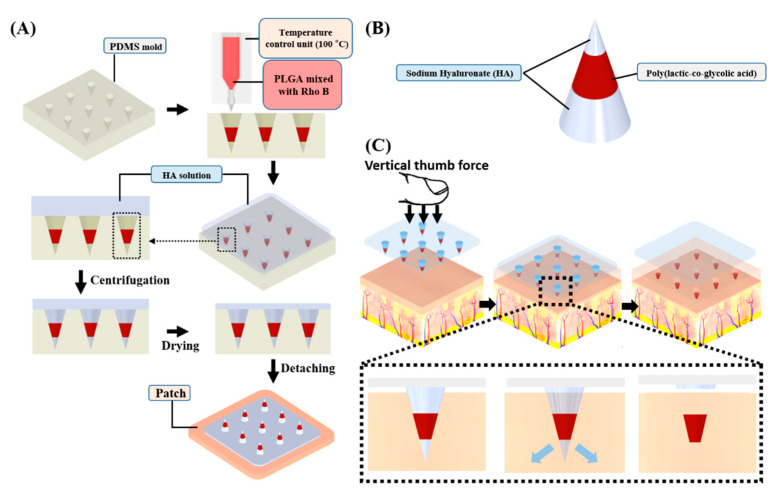
Schematic illustration of the fabrication process and application of an HMP-DMN patch. (**A**) Fabrication process using a PDMS mold. Heat-melted PLGA mixture loaded with rhodamine B was dispensed into the PDMS molds with the help of a dispenser robot and temperature control unit. Subsequently, the HA solution was cast into the PDMS mold, centrifuged, and dried. Finally, the HMP-DMN patch was detached from the PDMS mold. (**B**) Structure of an HMP-DMN. An HMP-DMN consists of three parts: HA—tip, PLGA mixture—middle, and HA—base layer. (**C**) Schematic representation of the application and separation of the HMP-DMNs from the patch. The HMP-DMN patch is inserted into the skin surface using vertical thumb force, following which the PLGA mixture separates from the patch upon dissolution of the HA base layer. The implanted PLGA mixture part then releases the drug inside the skin tissue in a sustained manner. (HMP-DMN: heat-melt PLGA dissolving microneedle, PDMS: polydimethylsiloxane, PLGA: poly(lactic-co-glycolic acid), HA: sodium hyaluronate).

Figure 1 shows the schematic fabrication process of the HMP-DMN patch via PLGA heat melting using a micromolding technique. The PDMS mold, which determines the shape of the DMN, was prepared by casting PDMS into the master structure. The liquid-state heated mixture of PLGA and Rho B was then dispensed into the mold cavities using a dispenser. The temperature control unit, which retains the liquid state of the PLGA mixture at 100 °C, was critical for fabricating the HMP-DMN patch without a solvent, because the DMN is shaped by the viscous liquid state. Owing to the thermoplastic property of PLGA, the heat-melted PLGA could fill the mold and compose the HMP-DMN patch without the need for toxic organic solvents.

However, the heated PLGA mixture coagulated in the PDMS mold after leaving the temperature control unit. This implies that it is difficult to maintain the liquid state of the PLGA mixture in the mold, and thus, filling the mold cavity completely with coagulated PLGA would hardly result in a sharp DMN. Therefore, HA solution, widely used as a backbone for DMN fabrication, was used as the second backbone polymer to fill the last remaining cavity of the mold and complete the DMN shape [27]. After the HA solution was poured into the mold containing the coagulated PLGA mixture droplet, centrifugation was performed to fill the entire cavity of the 3 × 3 array of microneedle-shaped molds. After drying the HA in the mold, the HMP-DMNs were detached from the mold, producing HMP-DMN patches. Because the two polymers were centrifuged in the same mold, the fabricated HMP-DMN had multiple polymer layers after drying. That is, the PLGA mixture, which is responsible for sustained release, was located at the center of the HMP-DMN, while the HA layers constituted the base and tip parts, as shown in Figure 1. Because the aqueous solution of HA had a relatively low viscosity compared to the coagulated PLGA, the HA solution could fill the tip part instead of PLGA after centrifugation, making a sharp tip structure that enabled successful skin penetration. In addition, the base layer of the DMN was also filled with HA and dissolved rapidly after skin penetration, upon contact with interstitial fluid in the skin tissue.

This novel method was able to fabricate HMP-DMN patches without the use of organic solvents by leveraging the thermoplastic property of PLGA. This indicates that therapeutics with thermostability, such as nifedipine and theophylline, can be encapsulated into HMP-DMNs [30,31]. This method also enabled the separation of HMP-DMNs from the patches, because HA, which completely dissolved rapidly after insertion into the skin, was located at the base of the HMP-DMN, connecting the PLGA mixture and the patch [32]. This separation property of the DMN is required for sustained delivery without the need to wear the patch, to overcome the problem of long-term attachment.

A schematic of the transdermal delivery of the HMP-DMNs is shown in Figure 1B. PLGA and HA were selected as materials for the HMP-DMN patch, because these biodegradable polymers are biocompatible and have enough mechanical strength for penetration into the skin [33]. HA, which is a hydrophilic polymer, has high solubility in interstitial fluid, whereas PLGA is a hydrophobic polymer [26,27]. In the HMP-DMN patch developed in this study, PLGA, which is responsible for the sustained drug release, was located in the middle of the HMP-DMN between HA layers. The HA layer located at the base enabled the HMP-DMNs to separate from the patch, due to its high solubility in the interstitial fluid, while the other layer constituted a sharp tip to enable penetration into the skin. Therefore, the HMP-DMN patch could separate the PLGA mixture from the patch by the dissolution of HA after penetration of the HMP-DMNs into the skin. After the HMP-DMN patch was inserted into the skin by vertical thumb force, HA at the base area of HMP-DMNs was entirely dissolved upon coming in contact with the body fluid, while the PLGA mixture, which has low solubility of body fluid, remained embedded below the skin and slowly released the encapsulated therapeutics [34]. According to this schematic illustration, the patch was detached without an external stimulus after HA dissolved in the base area. Using this design, the user has to wear a patch for only a few minutes, after which the encapsulated therapeutics are released for several weeks thereafter [24]. The release profile can be optimized by controlling the ratio of lactic acid and glycolic acid in the PLGA mixture, because the degradation rate of PLGA varies depending on the copolymer ratio [26]. The HMP-DMN patch also allows for patient compliance through self-administration with minimal training and rapid separation within 10 min.

### 3.2. Effect of PLGA Volume on the HMP-DMNs

**Figure 2:**
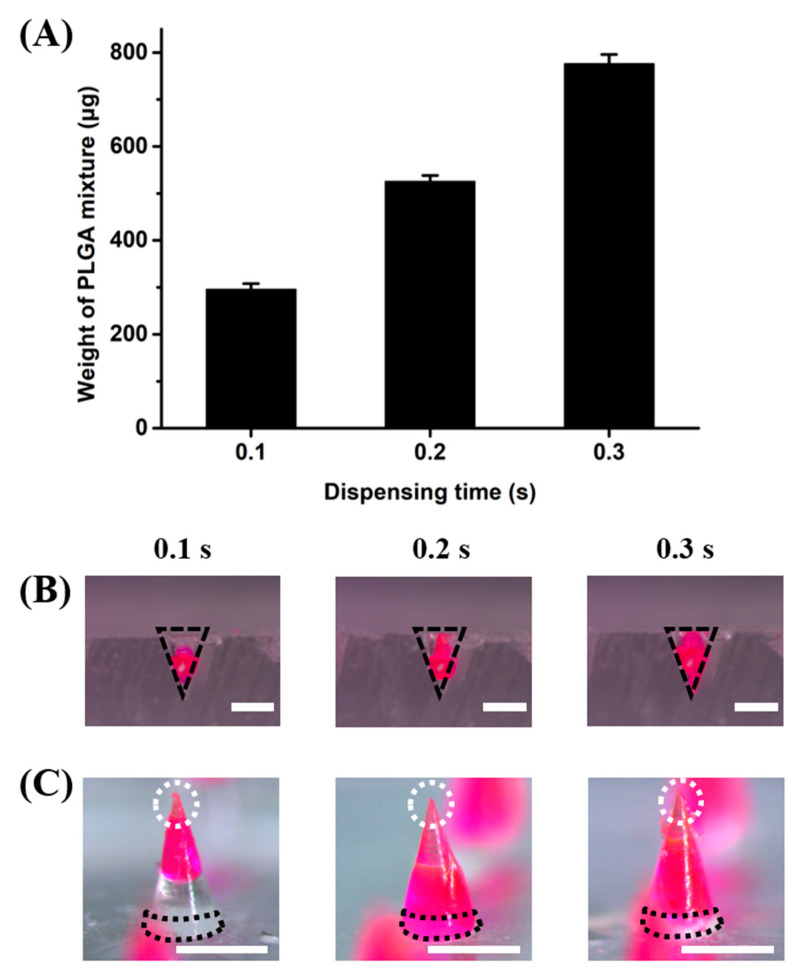
The amounts of PLGA mixtures in the molds at different dispensing times. (**A**) The amounts of PLGA mixtures increased as the dispensing time increased, in the order of 0.1, 0.2, and 0.3 s. (**B**) Microscopic images of the PLGA mixtures after they were dispensed into the molds (black dashed line: shape of the hole in the mold). The volume of the PLGA mixture increased as dispensing time increased. Scale bar: 500 µm. (**C**) Microscopic images of HMP-DMNs fabricated with different dispensing times of the PLGA mixture (0.1, 0.2, and 0.3 s) (dotted white circles: tip area; dotted black line: base area). Only the group with a dispensing time of 0.1 s had a base area that consisted of only HA. Scale bar: 500 µm. (PLGA: poly(lactic-co-glycolic acid), HMP-DMN: heat-melt PLGA dissolving microneedle, HA: sodium hyaluronate).

In HMP-DMNs, the amount of the PLGA mixture was controlled by altering the time taken for dispensing the PLGA mixture into the mold. The amount of PLGA would affect the geometry of the HMP-DMNs, consequently changing the composition of the tip, middle, and base layers. Therefore, weights of the PLGA mixture in the 3 × 3 array molds at different dispensing times of 0.1, 0.2, and 0.3 s were analyzed to optimize the final HMP-DMN geometry. As shown in Figure 2A, the weight of the PLGA mixture increased as the dispensing time increased, with weights of 296.7 ± 12.0 µg, 526.7 ± 12.0 µg, and 776.7 ± 20.0 µg (*n* = 3, mean ± SEM) for dispensing times of 0.1, 0.2, and 0.3 s, respectively. Because the volume of the PLGA mixture was determined by the amount of the PLGA mixture, which in turn depended on the dispensing time, the microscope images of the PLGA mixture occupying the conical molds were analyzed, as shown in Figure 2B, at the three different dispensing times. The black line indicates the shape of the mold. As expected, the PLGA mixture occupied more space from the tip of the mold as the amount of the PLGA mixture increased. This indicated that the dispensed PLGA mixture occupied the area near the tip first, and as the volume increased upon further dispensing, it filled the mold towards its base area, without significant change in the occupying volume at the tip area.

The configuration of the PLGA mixture was also analyzed at different dispensing times after the fabrication of the HMP-DMNs (Figure 2C). After the PLGA mixture was dispensed, the HA solution was cast in a mold and centrifuged at 3470× *g* for 30 min to completely fill the cavity, including the tip and base area. As expected, the PLGA mixture occupied the base of the HMP-DMNs with dispensing times of 0.2 and 0.3 s, while the base area of HMP-DMN with a dispensing time of 0.1 s consisted of HA. As described in the previous section, the presence of HA at the base was important for inducing the separation of HMP-DMNs after skin penetration for sustained delivery. The ideal height of HA at the base area would be over 250 µm, to meet the body fluid and dissolve after insertion, because DMN application with thumb force allows for only incomplete penetration into the skin, due to the shape of the DMN and skin elasticity [35]. Therefore, the HMP-DMN patch with a PLGA mixture dispensing time of 0.1 s allowed for the separation of the PLGA mixture from the patch when the HA layer dissolved after skin insertion, because the base area of this DMN consisted of only HA, as represented using a black dotted line (Figure 2C). However, in contrast to the groups with a dispensing time of 0.1 s, the base areas in HMP-DMN patch groups with PLGA mixture dispensing times of 0.2 and 0.3 s were occupied by PLGA.

### 3.3. Effect of Centrifugation Time on the HMP-DMNs

In the HMP-DMN fabrication method, the centrifugation step is important to fill the cavity of the entire mold with the PLGA mixture and HA. The effect of centrifugation time (10, 20, and 30 min), at the same centrifugation force of 3470× *g*, on the shape of the HMP-DMNs was evaluated at three different dispensing times with different amounts of PLGA mixture. The centrifugal force is the same regardless of the centrifugation time; however, the change in momentum is different depending on the centrifugation time, which means that the movement of HA in the mold is different depending on the centrifugation time.

**Figure 3:**
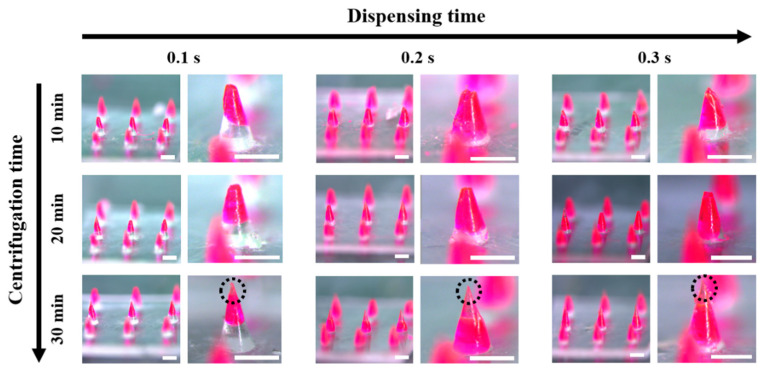
Images of HMP-DMN arrays at each fabrication condition. Sharpness increased depending on centrifugation time, in the order of 10, 20, and 30 min. In addition, the volume of the PLGA mixture increased as the dispensing time increased, at the same centrifugation time. The HMP-DMNs displayed the sharpest tips after 30 min of centrifugation time, when the volume of the PLGA mixture loaded with the model drug was controlled by tuning the dispensing time. Scale bar: 500 µm. (HMP-DMN: heat-melt PLGA dissolving microneedle, PLGA: poly(lactic-co-glycolic acid)).

As shown in Figure 3, HMP-DMNs fabricated with centrifugation times of 10 and 20 min had stubby tips, while the HMP-DMNs fabricated with a centrifugation time of 30 min had sharp tips, regardless of dispensing time (tips have been indicated using black dashed circle lines). This implied that centrifugation time had an effect on the filling of HA at the tip part of the mold instead of the coagulated PLGA because of the relatively low viscosity of HA compared to PLGA at room temperature. The sharp tip is shown only in the 30 min group with transparent HA, which indicates that a 30 min centrifugation time is necessary to fill the cavity in the mold. Correlating this result with the dispensing time of PLGA, which was optimized in the previous section, the geometry of HMP-DMNs was significantly affected by the amount of the PLGA mixture and the centrifugation time at the DMN fabrication step. Because DMN geometry is critical for skin penetration and successful implantation of the drug-encapsulated parts, optimization of the HMP-DMNs was further conducted with in vitro pig cadaver skin.

### 3.4. Skin Penetration of the HMP-DMN Patches

Previous studies have shown that the geometry of microneedles is significantly related to the skin penetration function, which is a critical factor for successful transdermal drug delivery [36,37]. The sharpness of the HMP-DMN tips and various configurations of the PLGA mixture and HA in the HMP-DMN patches could be controlled by optimizing the centrifugation and dispensing times, respectively, as shown in Figure 2 and Figure 3. The skin penetration test was performed using ex vivo porcine skin and Trypan Blue solution with fabricated HMP-DMNs, at different amounts of the PLGA mixture and centrifugation times.

**Figure 4:**
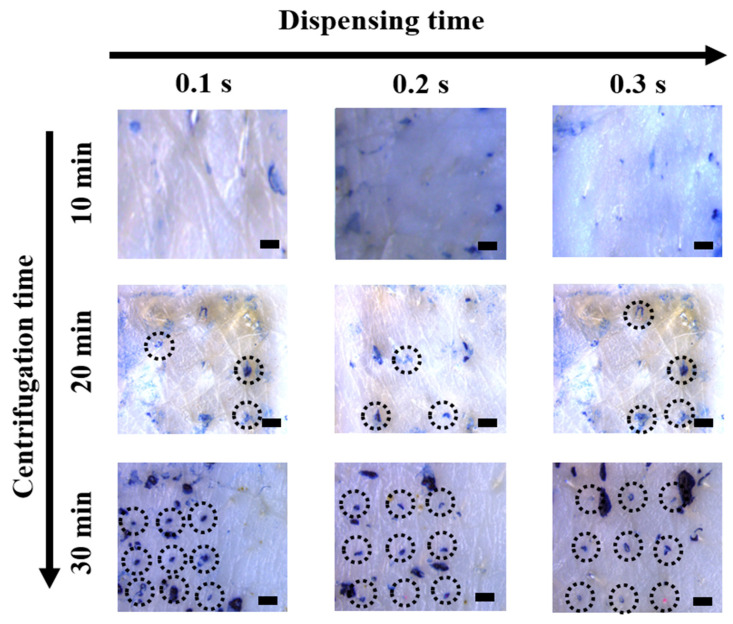
Microscope images of porcine skin penetrated by HMP-DMN patches (3 × 3 arrays) of different fabrication conditions. The HMP-DMN patches fabricated with a centrifugation time of 30 min successfully penetrated the skin, regardless of the dispensing time of the PLGA mixture, while HMP-DMN patches fabricated with centrifugation times of 10 min and 20 min made imperfect perforations. The dotted black circles indicate successfully penetrated perforations. Scale bar: 500 µm. (HMP-DMN: heat-melt PLGA dissolving microneedle, PLGA: poly(lactic-co-glycolic acid)).

As shown in Figure 4, HMP-DMNs fabricated with a centrifugation time of 10 min made no stained perforation, while the HMP-DMNs fabricated with a centrifugation time of 20 min penetrated 3.3 ± 0.7 of the 9 holes (*n* = 3, mean ± SEM) on the porcine skin. The HMP-DMN patch, which had a stubby tip due to insufficient centrifugation time, did not penetrate the porcine skin. In contrast, the HMP-DMN patches fabricated with a centrifugation time of 30 min were able to make 3 × 3 arrays of stained perforations on the porcine skin (Figure 4; dotted black circles), regardless of the dispensing time. This confirms that the HMP-DMN patch group fabricated with a centrifugation time of 30 min had sufficient time to make sharp tips for all the microneedles in the 3 × 3 arrays by filling the tip area in the mold with the HA solution, unlike the groups that were fabricated with centrifugation times of 10 and 20 min.

### 3.5. Morphological and Physical Properties of the HMP-DMN

**Figure 5:**
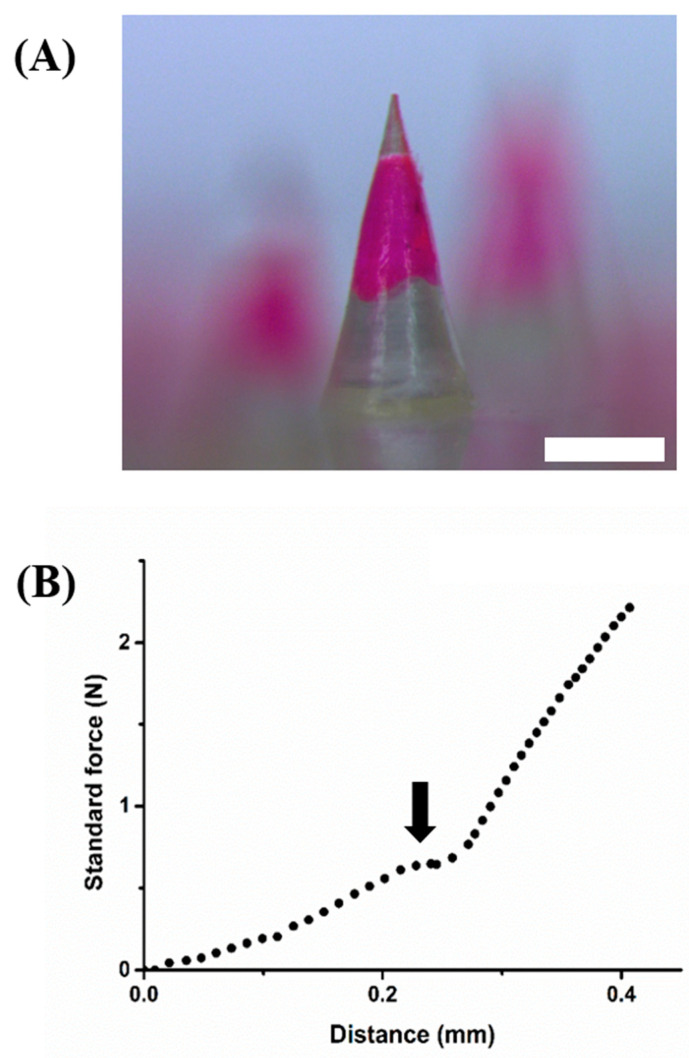
Morphological and mechanical properties of the HMP-DMNs. (**A**) Microscope image of a single HMP-DMN. (**B**) Fracture force of the HMP-DMN. The peak in the graph represents the fracture force of a single HMP-DMN. Scale bar: 500 µm. (HMP-DMN: heat-melt PLGA dissolving microneedle).

After optimization of the fabrication method of the HMP-DMN patch, depending on dispensing time of the PLGA mixture and centrifugation time, the final configuration was selected to HMP-DMN fabricated with a 0.1 s PLGA mixture dispensing time and 30 min centrifugation time. The morphologies of the selected HMP-DMNs were evaluated using a stereomicroscope and a digital microscope camera. Because the HMP-DMNs were fabricated using the molding casting method, the height and base diameter of the HMP-DMNs were similar to those of the master structures, i.e., 802.5 ± 3.1 µm and 450.3 ± 4.2 µm, respectively (Figure 5A). The lowest part of the PLGA mixture was located at 284 ± 4.6 µm above the base of the microneedle, while the height of the PLGA mixture was 337 ± 7.3 µm (*n* = 4, mean ± SEM). This means that the PLGA mixture could be completely inserted below the skin surface, even in the situation of incomplete insertion of the base layer, which has been criticized as an important issue in DMN application by various previous studies [11,38,39].

Although the optimized morphologies of the HMP-DMNs were partially composed of HA as the tip, the sharpness of the tip part of the structures was responsible for skin penetration. Therefore, the mechanical strength of the tip and its fracture force were analyzed. As shown in Figure 5B as a representative result, the force increased as the probe of the force machine pressed in the axial down, and the fractures occurred at the peak of the graph (0.69 N). Previous research has shown that the minimum fracture force for skin penetration is 0.058 N, indicating that the mechanical strength of the HMP-DMN was sufficient to penetrate the skin, in parallel with the skin perforation data shown in Figure 4 [40].

### 3.6. Separation of the HMP-DMN Patches

**Figure 6:**
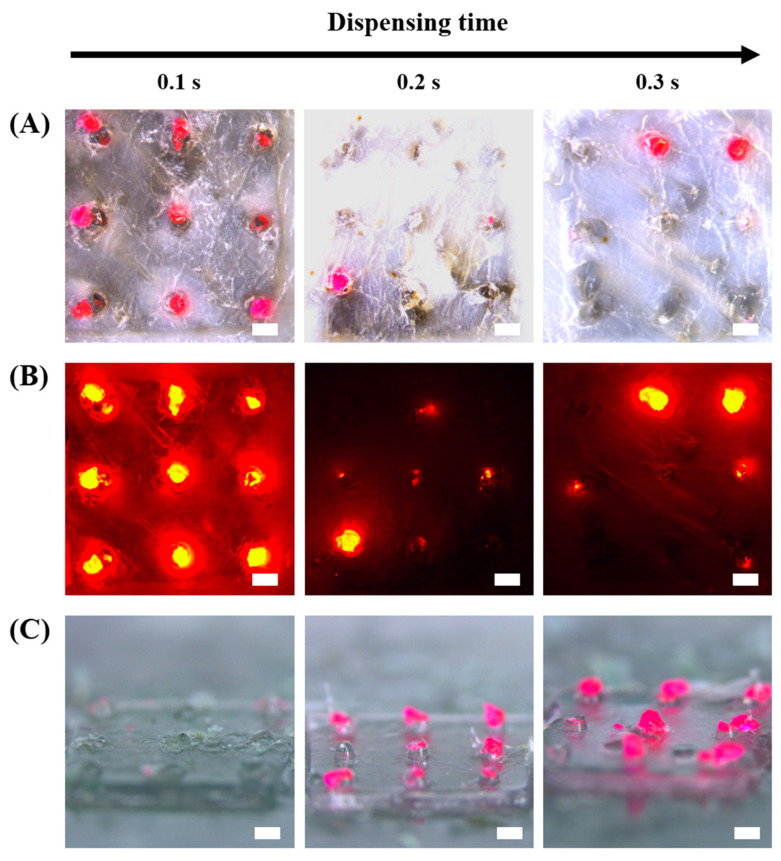
Separation of the PLGA part from the HMP-DMN patch at different PLGA dispensing times. After HMP-DMN patch application and detachment from pig cadaver skin, the (**A**) bright-field images of pig skin, (**B**) fluorescence images of pig skin, and (**C**) residual images of patches were obtained at different dispensing times of the PLGA mixture. Only the HMP-DMN patch with a dispensing time of 0.1 s showed complete insertion and separation of the PLGA mixture from the patch into the skin. All the HMP-DMN patches were fabricated at a centrifugation time of 30 min. Scale bar: 500 µm. (PLGA: poly(lactic-co-glycolic acid), HMP-DMN: heat-melt PLGA dissolving microneedle).

Usually, DMNs are fabricated on the patch, applied using thumb force, retained in the position until the DMNs fully dissolve, and then removed. In the case of sustained release, however, the DMN has a long-term attaching problem, because it dissolves for a long period. Therefore, it is necessary to separate the DMN from the patch to overcome this problem. Various rapidly separable DMNs have been designed by introducing bubbles and fabricating different materials that have high solubility at the base of the DMN [24,41]. In the HMP-DMN patches developed in this study, HA was designed to be located at the base, to allow for the separation of the HMP-DMNs, because HA has high solubility in interstitial fluid [42]. However, as described in the previous section, DMN application with thumb force resulted in incomplete penetration into the skin due to the shape of the DMN and skin elasticity. This implied that the base part under 250 µm was not able to penetrate the skin [35]. Therefore, the height of HA at the base was designed to meet the body fluid and, thus, dissolve quickly when inserted into the skin, while considering incomplete insertion. The height of HA at the base area depended on the location of the PLGA mixture in the HMP-DMN, because the PLGA mixture was first dispensed in the mold, and then HA was added to the mold. To evaluate the possibility of separation depending on the amount of PLGA, separation of the HMP-DMN patch with three different dispensing times of the PLGA mixture (0.1, 0.2, and 0.3 s) was analyzed. As shown in Figure 2 and Figure 3, the height of the HA base layer was only affected by the amount of PLGA mixture rather than the centrifugation time, because the coagulated PLGA mixture occupied the tip layer first and filled the base layer only as the volume increased. The centrifugation time for making the HMP-DMN patch was fixed at 30 min to make sharp DMNs with the ability to penetrate the skin (Figure 4). The application time was set at 10 min, for sufficient dissolution of the HA base layer and separation from the patch. As a result, based on the microscopic images (Figure 6A), fluorescence images (Figure 6B), and residual patch observations (Figure 6C), the PLGA layer in the HMP-DMN patch with a dispensing time of 0.1 s completely separated from the patch, unlike other groups that had an insufficient height of the HA base layer. Figure 6A,B shows that all the microneedles in the HMP-DMN array fabricated with a dispensing time of 0.1 s completely implanted into the skin, while only a few microneedles in the arrays fabricated with dispensing times of 0.2 and 0.3 s implanted into the skin. This result is related to the separation ability of the HMP-DMN patch after skin penetration, as shown in Figure 6C; only the HMP-DMN patch with a dispensing time of 0.1 s left no residual PLGA middle layer in the patch after application. Correlating these results, as dispensing time of the PLGA mixture increases, the PLGA layers occupy larger proportions, and the height of the HA base layer becomes insufficient for separation from the patch. In addition, to certify complete implantation into skin tissue of the PLGA mixture from HMP-DMNs with a dispensing time of 0.1 s, sectional images were obtained using a fluorescence microscope. As shown in Figure S1, PLGA mixtures in arrays with a dispensing time of 0.1 s remained below the skin after separation from the patch, which implies complete implantation of the PLGA mixture into the skin tissue. It was confirmed that the amount of the PLGA mixture was related to HMP-DMN separation and the group with a dispensing time of 0.1 s was able to be completely inserted into the skin surface and detach.

**Figure 7:**
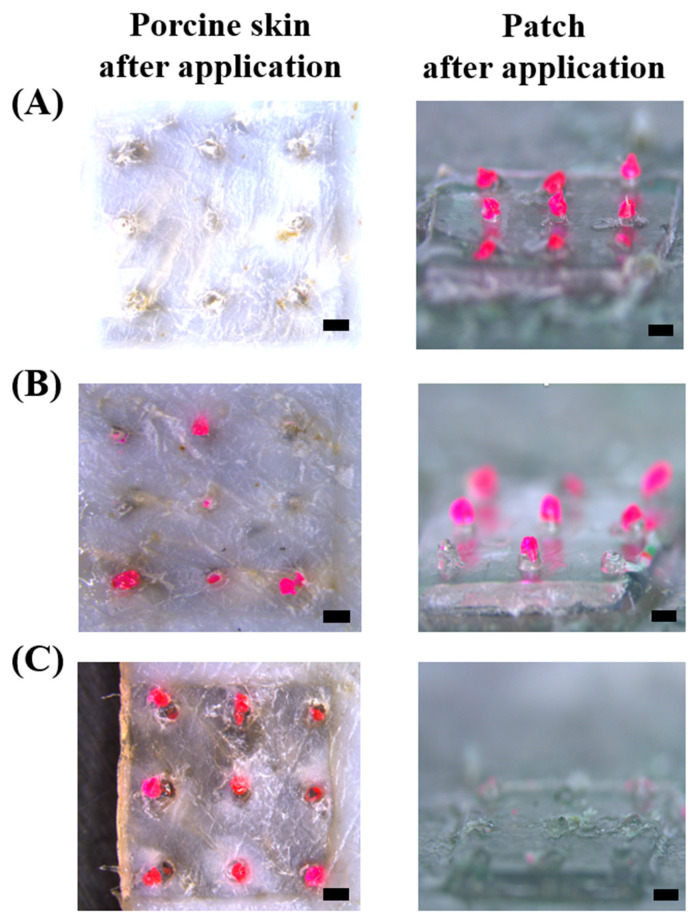
Separation of the PLGA part from the HMP-DMN patch at different application times. Microscopic images of porcine skin and HMP-DMN patches at different application times: (**A**) 0 min, (**B**) 5 min, and (**C**) 10 min after insertion. The left column shows porcine skin after application, and the right column shows HMP-DMN residuals on the patch after application. After application for 10 min, the PLGA mixture from the HMP-DMN patch was found to have been completely implanted into the skin. Scale bar: 500 µm. (PLGA: poly(lactic-co-glycolic acid), HMP-DMN: heat-melt PLGA dissolving microneedle).

To evaluate the separation time of the HMP-DMN patch with a dispensing time of 0.1 s, separation analysis was performed at different application times. The HMP-DMN patch was pressed by thumb force into pig cadaver skin and then detached from the skin after 0, 5, and 10 min, because HA has been shown to completely dissolve 10 min after skin insertion [35]. As shown in Figure 7A, none of the HMP-DMNs separated from the patch, and HMP-DMN remained on the patch when it was immediately detached after application (0 min of the application group). After 5 min, two of the HMP-DMNs from the 3 × 3 array were found to have been entirely inserted into the skin (Figure 7B), because every needle in the array had a base area composed of HA of different heights. However, at 10 min, all the HMP-DMNs from the 3 × 3 arrays were found to have fully separated from the patch, with no residuals remaining on the patch (Figure 7C). This means that the patients would have to wear the HMP-DMN patch for just 10 min after insertion, after which the patch can be detached, and the PLGA mixture will keep releasing therapeutics below the skin without the patch. In view of this result, this system can possibly overcome the limitations of traditionally implanted DMNs, and thus, can improve patient compliance without attaching problems. Further studies focused on skin pore closure after DMN penetration and complete implantation of DMN should also be conducted to achieve the successful utilization of HMP-DMN technology [43,44].

### 3.7. Release Profile of Rho B

**Figure 8:**
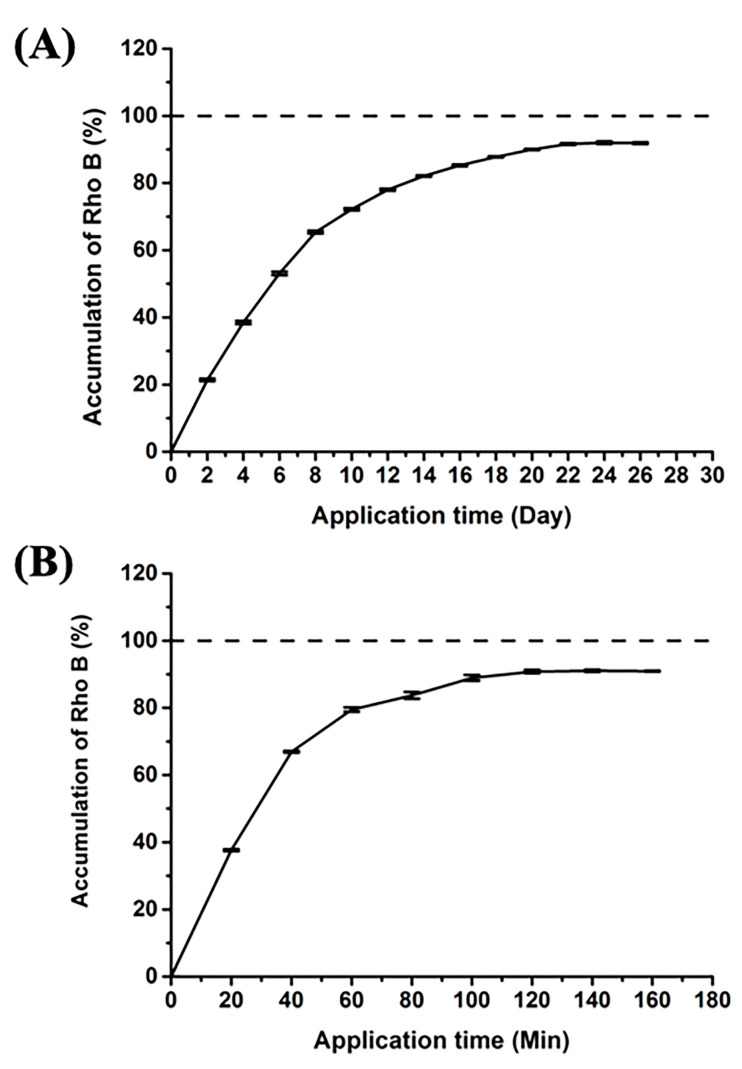
Release of Rho B from the HMP-DMN and control DMN patches. (**A**) Here, 90% of Rho B was released within 26 days. (**B**) Here, 90% of Rho B was released within 160 min. (Rho B: rhodamine B, HMP-DMN: heat-melt PLGA dissolving microneedle, DMN: dissolving microneedle).

Rho B was loaded into the HMP-DMN patch, to quantitatively demonstrate the delivery of Rho B via HMP-DMNs using PBS containing 25% (*v/v*) ethanol as a medium [29]. The release profile was analyzed to estimate the release period of the HMP-DMN patch, which encapsulated 2.97 µg of Rho B as a model dye (1% (*w/w*) in 296.7 ± 12.0 µg of PLGA mixture), as compared to Rho B from the control DMN patch without PLGA, as shown in Figure 8. This graph shows that 90% of Rho B from the HMP-DMN patch was released within 26 days, while the control released 90% of Rho B within 160 min.

The results for the drug release profiles indicated that the HMP-DMN patch enabled the release of Rho B sustainably and could achieve a delivery period of nearly four weeks. Therefore, HMP-DMNs can be used for the delivery of long-acting therapeutics that require frequent medication with strict cycles, such as contraceptive hormone and high blood pressure medicine, to overcome the problem of frequent application. Although optimization of the improved DMN patch fabrication method for sustained drug delivery and enhanced user compliance was carried out in this study, further research is required for practical development. Encapsulation of sufficient human doses of therapeutics in HMP-DMN arrays with optimized DMN geometry and enlarged array for practical purposes should be attempted. After successful future studies that investigate in vivo drug release profiles of therapeutics that require sustained release profiles, such as levonorgestrel, ibuprofen, or progesterone, a convenient and effective medication system could be developed using the DMN technology.

## 4. Conclusions

Novel HMP-DMN patches composed of biocompatible polymers, HA and PLGA, were fabricated for sustained drug release to overcome the limitations of previous studies on the inevitable use of organic solvents. PLGA was melted by heat, homogenized with therapeutics, and placed in the middle of the HMP-DMN, while HA provided a sharp tip for skin penetration and rapid separation from the patch. The morphology of the HMP-DMN was optimized for various dispensing times of the PLGA mixture and centrifugation times during the DMN fabrication process. Optimized HMP-DMNs displayed successful skin penetration, rapid separation, and sustained drug release for 4 weeks. The resulting HMP-DMN patches would be a possibly innovative drug delivery system for heat-tolerable drugs that require long-acting efficacy, accompanied by further studies including evaluation of drug activity after the heating process. In addition, this novel multilayered HMP-DMN patch fabricated using a simple process without an organic solvent will allow for the innovative utilization of PLGA as an alternative matrix polymer for DMNs.

## Data Availability

Not applicable.

## Supplementary Materials

The following are available online at https://www.mdpi.com/article/10.3390/pharmaceutics13071058/s1, Figure S1: Fluorescence image of sectioned pig skin after HMP-DMN patch application.

## References

[1] Larrañeta E., McCrudden M.T., Courtenay A.J., Donnelly R.F. (2016). Microneedles: A new frontier in nanomedicine delivery. Pharm. Res..

[2] Prausnitz M.R. (2004). Microneedles for transdermal drug delivery. Adv. Drug Deliv. Rev..

[3] Shin C.I., Jeong S.D., Rejinold N.S., Kim Y.C. (2017). Microneedles for vaccine delivery: Challenges and future perspectives. Ther. Deliv..

[4] Dangol M., Yang H., Li C.G., Lahiji S.F., Kim S., Ma Y., Jung H. (2016). Innovative polymeric system (IPS) for solvent-free lipophilic drug transdermal delivery via dissolving microneedles. J. Control. Release.

[5] Nayak A., Das D.B. (2013). Potential of biodegradable microneedles as a transdermal delivery vehicle for lidocaine. Biotechnol. Lett..

[6] Ito Y., Ohta J., Imada K., Akamatsu S., Tsuchida N., Inoue G., Inoue N., Takada K. (2013). Dissolving microneedles to obtain rapid local anesthetic effect of lidocaine at skin tissue. J. Drug Target..

[7] Kim Y.C., Park J.H., Prausnitz M.R. (2012). Microneedles for drug and vaccine delivery. Adv. Drug Deliv. Rev..

[8] Vassilieva E.V., Kalluri H., McAllister D., Taherbhai M.T., Esser E.S., Pewin W.P., Pulit-Penaloza J.A., Prausnitz M.R., Compans R.W., Skountzou I. (2015). Improved immunogenicity of individual influenza vaccine components delivered with a novel dissolving microneedle patch stable at room temperature. Drug Deliv. Transl. Res..

[9] Duong H.T.T., Yin Y., Thambi T., Kim B.S., Jeong J.H., Lee D.S. (2020). Highly potent intradermal vaccination by an array of dissolving microneedle polypeptide cocktails for cancer immunotherapy. J. Mater. Chem. B.

[10] Frew P.M., Paine M.B., Rouphael N., Schamel J., Chung Y., Mulligan M.J., Prausnitz M.R. (2020). Acceptability of an inactivated influenza vaccine delivered by microneedle patch: Results from a phase I clinical trial of safety, reactogenicity, and immunogenicity. Vaccine.

[11] Ling M.H., Chen M.C. (2013). Dissolving polymer microneedle patches for rapid and efficient transdermal delivery of insulin to diabetic rats. Acta Biomater..

[12] Ita K. (2017). Dissolving microneedles for transdermal drug delivery: Advances and challenges. Biomed. Pharmacother..

[13] Eum J., Kim Y., Um D.J., Shin J., Yang H., Jung H. (2021). Solvent-Free Polycaprolactone Dissolving Microneedles Generated via the Thermal Melting Method for the Sustained Release of Capsaicin. Micromachines.

[14] Park J.H., Allen M.G., Prausnitz M.R. (2006). Polymer microneedles for controlled-release drug delivery. Pharm. Res..

[15] He M., Yang G., Zhao X., Zhang S., Gao Y. (2020). Intradermal implantable PLGA microneedles for etonogestrel sustained release. J. Pharm. Sci..

[16] Nguyen H.X., Banga A.K. (2018). Delivery of methotrexate and characterization of skin treated by fabricated PLGA microneedles and fractional ablative laser. Pharm. Res..

[17] Zhao X., Zhang S., Yang G., Zhou Z., Gao Y. (2020). Exploring Trehalose on the release of levonorgestrel from implantable PLGA microneedles. Polymers.

[18] Kim J.D., Kim M., Yang H., Lee K., Jung H. (2013). Droplet-born air blowing: Novel dissolving microneedle fabrication. J. Control. Release.

[19] Lee J.W., Choi S.O., Felner E.I., Prausnitz M.R. (2011). Dissolving microneedle patch for transdermal delivery of human growth hormone. Small.

[20] Yang H., Kim S., Kang G., Lahiji S.F., Jang M., Kim Y.M., Kim J.M., Cho S.N., Jung H. (2017). Centrifugal lithography: Self-shaping of polymer microstructures encapsulating biopharmaceutics by centrifuging polymer drops. Adv. Healthc. Mater..

[21] Bala I., Hariharan S., Kumar M.R. (2004). PLGA nanoparticles in drug delivery: The state of the art. Crit. Rev. Ther. Drug Carrier Syst..

[22] Paul D., Dey T.K., Dhar P., Ficai D., Grumezescu A.M. (2017). Nanoformulation and administration of PUFA-rich systems for applications in modern healthcare. Nanostructures for Novel Therapy.

[23] Jenkins M.J., Harrison K.L. (2006). The effect of molecular weight on the crystallization kinetics of polycaprolactone. Polym. Adv. Technol..

[24] Li W., Terry R.N., Tang J., Feng M.R., Schwendeman S.P., Prausnitz M.R. (2019). Rapidly separable microneedle patch for the sustained release of a contraceptive. Nat. Biomed. Eng..

[25] McCrudden M.T., Alkilani A.Z., McCrudden C.M., McAlister E., McCarthy H.O., Woolfson A.D., Donnelly R.F. (2014). Design and physicochemical characterisation of novel dissolving polymeric microneedle arrays for transdermal delivery of high dose, low molecular weight drugs. J. Control. Release.

[26] Ji W., Yang F., Seyednejad H., Chen Z., Hennink W.E., Anderson J.M., van den Beucken J.J., Jansen J.A. (2012). Biocompatibility and degradation characteristics of PLGA-based electrospun nanofibrous scaffolds with nanoapatite incorporation. Biomaterials.

[27] Liu S., Jin M.N., Quan Y.S., Kamiyama F., Katsumi H., Sakane T., Yamamoto A. (2012). The development and characteristics of novel microneedle arrays fabricated from hyaluronic acid, and their application in the transdermal delivery of insulin. J. Control. Release.

[28] Kim S., Eum J., Yang H., Jung H. (2019). Transdermal finasteride delivery via powder-carrying microneedles with a diffusion enhancer to treat androgenetic alopecia. J. Control. Release.

[29] Wang S.H., Zhang L.C., Lin F., Sa X.Y., Zuo J.B., Shao Q.X., Chen G.S., Zeng S. (2005). Controlled release of levonorgestrel from biodegradable poly (D, L-lactide-co-glycolide) microspheres: In vitro and in vivo studies. Int. J. Pharm..

[30] Forster A., Hempenstall J., Rades T. (2001). Characterization of glass solutions of poorly water-soluble drugs produced by melt extrusion with hydrophilic amorphous polymers. J. Pharm. Pharmacol..

[31] Young C.R., Dietzsch C., Cerea M., Farrell T., Fegely K.A., Rajabi-Siahboomi A., McGinity J.W. (2005). Physicochemical characterization and mechanisms of release of theophylline from melt-extruded dosage forms based on a methacrylic acid copolymer. Int. J. Pharm..

[32] Kang G., Tu T.N.T., Kim S., Yang H., Jang M., Jo D., Ryu J., Baek J., Jung H. (2018). Adenosine-loaded dissolving microneedle patches to improve skin wrinkles, dermal density, elasticity and hydration. Int. J. Cosmet. Sci..

[33] Liu S., Jin M.N., Quan Y.S., Kamiyama F., Kusamori K., Katsumi H., Sakane T., Yamamoto A. (2014). Transdermal delivery of relatively high molecular weight drugs using novel self-dissolving microneedle arrays fabricated from hyaluronic acid and their characteristics and safety after application to the skin. Eur. J. Pharm. Biopharm..

[34] Holy C.E., Dang S.M., Davies J.E., Shoichet M.S. (1999). In vitro degradation of a novel poly (lactide-co-glycolide) 75/25 foam. Biomaterials.

[35] Lee S., Fakhraei Lahiji S., Jang J., Jang M., Jung H. (2019). Micro-pillar integrated dissolving microneedles for enhanced transdermal drug delivery. Pharmaceutics.

[36] Davis S.P., Landis B.J., Adams Z.H., Allen M.G., Prausnitz M.R. (2004). Insertion of microneedles into skin: Measurement and prediction of insertion force and needle fracture force. J. Biomech..

[37] Li Y., Hu X., Dong Z., Chen Y., Zhao W., Wang Y., Zhang L., Chen M., Wu C., Wang Q. (2020). Dissolving Microneedle Arrays with Optimized Needle Geometry for Transcutaneous Immunization. Eur. J. Pharm. Sci..

[38] Chen M.C., Huang S.F., Lai K.Y., Ling M.H. (2013). Fully embeddable chitosan microneedles as a sustained release depot for intradermal vaccination. Biomaterials.

[39] Lahiji S.F., Dangol M., Jung H. (2015). A patchless dissolving microneedle delivery system enabling rapid and efficient transdermal drug delivery. Sci. Rep..

[40] Lee C., Kim H., Kim S., Lahiji S.F., Ha N.Y., Yang H., Kang G., Nguyen H.Y.T., Kim Y., Choi M.S. (2018). Comparative Study of Two Droplet-Based Dissolving Microneedle Fabrication Methods for Skin Vaccination. Adv. Healthc. Mater..

[41] DeMuth P.C., Garcia-Beltran W.F., Ai-Ling M.L., Hammond P.T., Irvine D.J. (2013). Composite dissolving microneedles for coordinated control of antigen and adjuvant delivery kinetics in transcutaneous vaccination. Adv. Funct. Mater..

[42] Brown M.B., Jones S.A. (2005). Hyaluronic acid: A unique topical vehicle for the localized delivery of drugs to the skin. J. Eur. Acad. Dermatol. Venereol..

[43] Haridass I.N., Wei J.C., Mohammed Y.H., Crichton M.L., Anderson C.D., Henricson J., Sanchez W.Y., Meliga S.C., Grice J.E., Benson H.A.E. (2019). Cellular metabolism and pore lifetime of human skin following microprojection array mediation. J. Control. Release.

[44] Wei J.C., Haridass I.N., Crichton M.L., Mohammed Y.H., Meliga S.C., Sanchez W.Y., Grice J.E., Benson H.A.E., Roberts M.S., Kendall M.A. (2018). Space-and time-resolved investigation on diffusion kinetics of human skin following macromolecule delivery by microneedle arrays. Sci. Rep..

