# Supplementary material for: Optimization of Layered Dissolving Microneedle for Sustained Drug Delivery Using Heat-Melted Poly(Lactic-Co-glycolic Acid)
Source: Pharmaceutics. 2021 Jul 10;13(7):1058. doi: 10.3390/pharmaceutics13071058 (PMC8309023; doi:10.3390/pharmaceutics13071058)
Supplement: Supplementary file 1 [file pharmaceutics-13-01058-s001.zip › pharmaceutics-1252247-SI.pdf]

pharmaceutics

MDPI

Supplementary Materials: Optimization of Layered Dissolving Microneedle for Sustained Drug Delivery using Heat-melted Poly(lactic-co-glycolic acid)

Chisong Lee, Jinkyung Kim, Daniel Junmin Um, Youseong Kim, Hye Su Min, Jiwoo Shin, Jee Hye Nam, Geonwoo Kang, Mingyu Jang, Huisuk Yang and Hyungil Jung

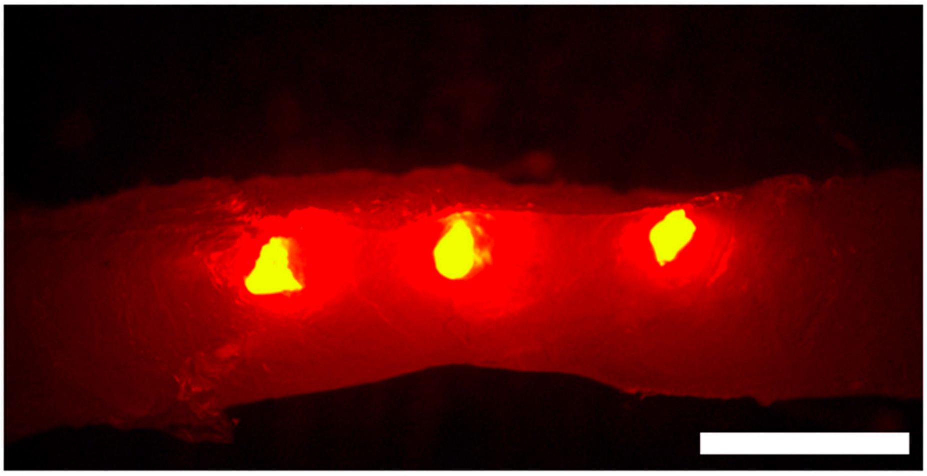

Figure S1. Fluorescence image of sectioned pig skin post HMP-DMN patch application**.** The HMP-DMN patch was fabricated with a dispensing time of 0.1 s and centrifugation time of 30 min. The PLGA mixture loaded with rhodamine B was successfully implanted inside the skin tissue. Scale bar: 1000 μm. (HMP-DMN: heat-melt PLGA dissolving microneedle, PLGA: poly(lactic-co-glycolic acid)).
